# Visual Dermatologic Case Report: Juvenile Xanthogranuloma of the Labia Majora in a Pediatric Patient

**DOI:** 10.1155/crpe/8841595

**Published:** 2026-05-28

**Authors:** Kelly Frasier, Silvija P. Gottesman, Pooja R. Shah

**Affiliations:** ^1^ Department of Dermatology, Northwell Health, New Hyde Park, New York, USA, northwell.edu; ^2^ Departments of Dermatology and Pathology, Northwell Health at Zucker School of Medicine, New Hyde Park, New York, USA

## Abstract

Juvenile xanthogranuloma (JXG), the most common non–Langerhans cell histiocytosis of childhood, typically presents as a benign, self‐limited cutaneous nodule on the head, neck, or trunk during early infancy. Genital involvement is exceedingly rare and poses distinct diagnostic challenges due to anatomic concealment and a broad differential diagnosis. A 5‐year‐old female presented with a solitary, well‐demarcated, tan‐brown, firm, dome‐shaped papule on the left labia majora that had been intermittently pruritic for approximately 1 month. Dermoscopic evaluation revealed a yellow hue, prompting referral for excisional biopsy. Histopathologic examination demonstrated a dense dermal infiltrate of foamy histiocytes with characteristic Touton giant cells and associated lymphocytes and eosinophils, without cytologic atypia or malignant features, confirming JXG. The patient’s dermatologic history included chronic atopic dermatitis, severe xerosis, pityriasis alba, and a stable café‐au‐lait patch, suggesting an underlying inflammatory cutaneous milieu. No additional lesions or systemic symptoms were identified. Ophthalmologic screening was performed in accordance with established recommendations and was unremarkable. Surgical excision was both diagnostic and curative, with no recurrence on follow‐up. This case highlights a rare vulvar presentation of JXG and emphasizes the importance of maintaining diagnostic vigilance for histiocytic disorders in pediatric patients with isolated, persistent dermal papules in uncommon locations. Visual documentation of this entity expands the recognized morphologic and anatomic spectrum of JXG, reinforces the role of biopsy in diagnostically uncertain genital lesions, and facilitates timely reassurance, appropriate surveillance, and prevention of unnecessary intervention.

## 1. Introduction

Juvenile xanthogranuloma (JXG), the most common non–Langerhans cell histiocytosis of childhood, classically presents as a benign, self‐limited cutaneous nodule localized to the head, neck, or trunk during early infancy. JXG most frequently occurs in infants and young children, with the majority of cases presenting within the first year of life, and is estimated to account for a significant proportion of pediatric non‐Langerhans histiocytic disorders, although precise population‐based prevalence data remain limited due to its typically benign and underreported course.

Involvement of the genital region remains exceedingly uncommon and introduces distinct diagnostic challenges, driven by anatomic concealment and an expansive differential diagnosis that includes both benign and malignant entities. Recognition of atypical presentations is therefore critical to avoid diagnostic delay, unnecessary intervention, and patient or caregiver distress.

The objective of this report is to describe a rare vulvar presentation of JXG in a pediatric patient, highlighting its clinical, dermoscopic, and histopathologic features, and to expand the existing literature on uncommon anatomic manifestations of this entity.

All procedures performed were conducted in accordance with institutional ethical standards. Informed consent for publication of clinical details and images was obtained from the patient’s legal guardian, and all identifying information has been appropriately de‐identified to ensure patient confidentiality.

## 2. Case Report

**FIGURE 1:**
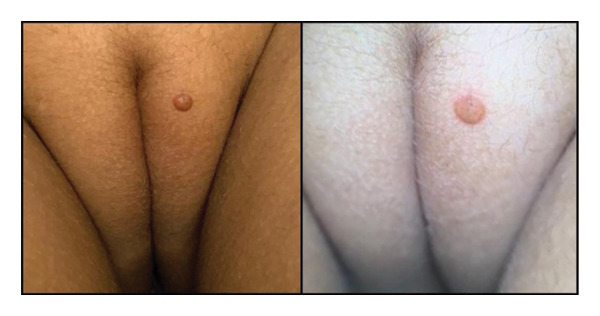
Well‐demarcated, tan‐brown, firm, dome‐shaped papule on the left labia majora of the vulva seen in a clinical photo (left) and dermoscopic image (right).

**FIGURE 2:**
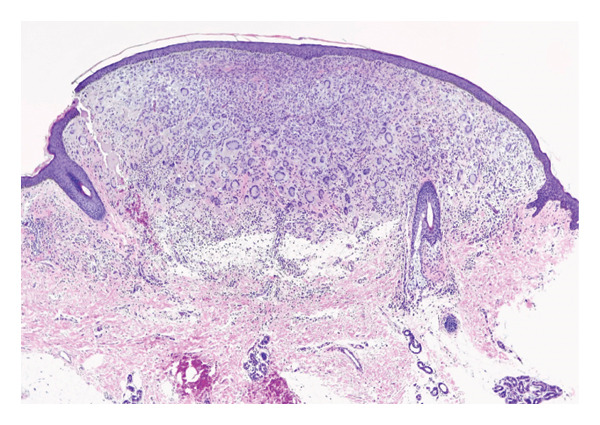
Dome‐shaped papule within the dermis comprised of histiocytes, giant cells, lymphocytes, and eosinophils (x100 magnification, H&E).

**FIGURE 3:**
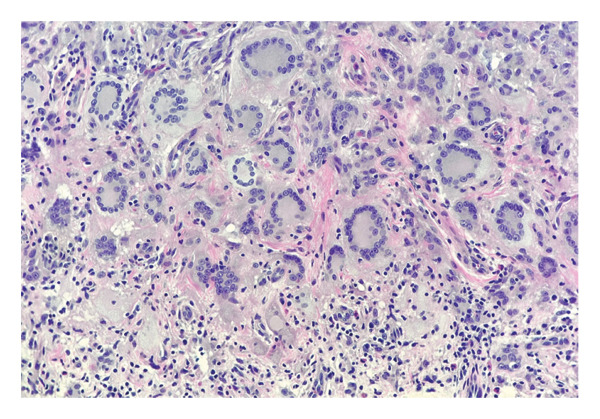
Close‐up view of the histiocytes, characteristic Touton giant cells, with background of lymphocytes and scattered eosinophils (x400 magnification, H&E).

A solitary, well‐demarcated, tan‐brown, firm, dome‐shaped papule involving the left labia majora of a 5‐year‐old female represented an uncommon anatomic manifestation of JXG, subsequently confirmed by histopathologic evaluation. The lesion was largely asymptomatic, with intermittent pruritus and excoriation reported over approximately 1 month (Figure 1). Dermoscopic assessment demonstrated a yellow hue consistent with the characteristic “setting sun” pattern, reflecting dermal accumulation of lipid‐laden histiocytes [1]. Referral to plastic surgery was undertaken for excisional biopsy; histopathologic analysis revealed a dense dermal infiltrate of foamy histiocytes with prominent Touton giant cells, accompanied by lymphocytes and eosinophils, without cytologic atypia or malignant features (Figures 2 and 3).

The patient’s dermatologic history was notable for chronic atopic dermatitis with marked xerosis, pityriasis alba, and a stable café‐au‐lait patch localized to the left neck, chest, and clavicular region, suggesting a complex and potentially dysregulated cutaneous inflammatory milieu. No additional lesions were identified on comprehensive skin examination, and no personal or family history of cutaneous malignancy or systemic histiocytic disease was elicited. Despite the absence of systemic symptoms, ophthalmologic evaluation was performed in accordance with established recommendations, given the potential for asymptomatic yet vision‐threatening ocular involvement. Ocular manifestations occur in fewer than 1% of cases but demonstrate increased prevalence among patients with multiple cutaneous lesions or early‐onset disease, reinforcing the importance of screening even in clinically asymptomatic presentations.

Surgical excision served both diagnostic and definitive therapeutic purposes. Follow‐up evaluation demonstrated appropriate wound healing without evidence of recurrence. No additional intervention was indicated, given the lesion’s limited size, benign histopathologic features, and absence of systemic involvement.

## 3. Discussion

JXG represents the most prevalent form of non–Langerhans cell histiocytosis in pediatric populations [2], yet vulvar involvement remains exceptionally rare [3]. Histopathologic characterization includes dermal proliferation of lipidized histiocytes with Touton giant cells and immunophenotypic positivity for CD68 and factor XIIIa, with absence of CD1a and langerin, facilitating distinction from Langerhans cell histiocytosis [4, 5]. Lesions most frequently arise on the head, neck, or trunk within the first year of life. Dermoscopic findings, including the characteristic “setting sun” appearance, reflect underlying lipid‐laden histiocytes within the dermis and may serve as a useful noninvasive diagnostic clue in clinically ambiguous presentations [1].

Solitary cutaneous lesions typically follow a benign and self‐limited course; however, genital presentations, particularly those involving the labia, introduce diagnostic complexity due to both rarity and a broad differential diagnosis encompassing infectious etiologies, melanocytic nevi, adnexal tumors, and Langerhans cell histiocytosis [6]. Large pediatric series support spontaneous regression in the majority of solitary lesions, reinforcing conservative management following diagnostic confirmation [7]. Delayed recognition may occur in anatomically concealed regions, underscoring the importance of comprehensive full‐body skin examination in pediatric dermatologic assessment.

Ophthalmologic screening remains an important consideration due to the potential for asymptomatic ocular involvement, which, although rare, may be vision‐threatening. Increased risk has been observed in patients with multiple lesions or early‐onset disease, supporting evaluation even in the absence of overt symptoms [8–10].

Reports of vulvar or genital JXG in the pediatric population remain limited, with documented cases frequently presenting as clinical mimickers of neoplastic or infectious processes [11, 12]. Published case reports describe a spectrum of presentations involving the labia majora, vulvar soft tissue, and adjacent genital structures, most commonly as solitary, firm, well‐circumscribed nodules prompting concern for malignancy. A case involving a 10‐year‐old child demonstrated a progressively enlarging, asymptomatic labial nodule ultimately diagnosed as JXG following excisional biopsy, with authors emphasizing the rarity of vulvar involvement and the predominance of similar reports in adult populations [13]. Another report described a 16‐month‐old female presenting with a solid vulvar mass initially concerning for rhabdomyosarcoma on imaging, underscoring the degree of diagnostic uncertainty associated with deep or atypical lesions in this region; histopathologic evaluation ultimately confirmed JXG, with no recurrence observed on follow‐up [3]. Additional rare presentations include involvement of clitoral connective tissue in early infancy, further expanding the anatomic spectrum of genital JXG and highlighting its ability to arise across diverse vulvar substructures [14].

Across reported cases, genital JXG most often presents as a solitary lesion and frequently raises concern for malignancy due to its location and morphology, prompting diagnostic biopsy. Histopathologic evaluation supports definitive diagnosis and guides management. The current case demonstrates close concordance with previously described presentations while further expanding the clinical and visual characterization of pediatric vulvar JXG within a limited but growing body of literature.

Several limitations warrant consideration. Findings are derived from a single patient, limiting generalizability and precluding definitive conclusions regarding the full clinical spectrum, natural history, or optimal management strategies for vulvar JXG. Follow‐up duration remains limited, restricting assessment of long‐term recurrence risk or delayed extracutaneous involvement. Although dermoscopic and histopathologic findings were characteristic, variability across reported cases, including differences in lesion depth, growth kinetics, and initial clinical suspicion, suggests potential heterogeneity in presentation that may complicate early recognition, particularly in anatomically concealed sites. Additionally, the rarity of reported cases introduces an inherent publication bias, with atypical or diagnostically challenging presentations more likely to be documented, potentially skewing the perceived clinical spectrum. Despite these limitations, careful integration of existing case reports supports the broader clinical implication that vulvar JXG, while rare, should remain within the differential diagnosis of pediatric genital lesions to facilitate timely diagnosis, appropriate reassurance, and avoidance of unnecessary invasive intervention.

## 4. Conclusion

Recognition of JXG in atypical anatomic locations expands the clinical spectrum of this entity and reinforces the necessity of maintaining diagnostic vigilance for histiocytic disorders in pediatric patients presenting with isolated, persistent dermal papules. Histopathologic confirmation remains essential in anatomically sensitive or diagnostically ambiguous sites, enabling appropriate reassurance, avoidance of unnecessary intervention, and timely evaluation for extracutaneous involvement.

## Funding

No funding was received for this manuscript.

## Consent

No written consent has been obtained from the patient as there are no patient identifiable data included in this case report.

## Conflicts of Interest

The authors declare no conflicts of interest.

## Data Availability

Data sharing is not applicable to this article as no datasets were generated or analyzed during the current study.
